# Catch-up Growth in Prepubertal Children Treated for Juvenile Hypothyroidism and Growth Hormone Deficiency can be Modelled with a Monomolecular Function

**DOI:** 10.4274/jcrpe.galenos.2020.2020.0130

**Published:** 2021-02-26

**Authors:** Jan M. Wit, Theo C. J. Sas, Michael B. Rank, Paula van Dommelen

**Affiliations:** 1 Leiden University Medical Center, Department of Paediatrics, Leiden, The Netherlands; 2 Sophia Children’s Hospital, University Medical Center Rotterdam, Department of Paediatric Endocrinology; National Diabetes Care and Research Center, Clinic of Diabetes, Rotterdam, The Netherlands; 3 University Children’s Hospital, Tübingen, Germany; 4 The Netherlands Organization for Applied Scientific Research TNO, Leiden, The Netherlands

**Keywords:** Growth, catch-up growth, coeliac disease, growth hormone deficiency, hypothyroidism

## Abstract

**Objective:**

We hypothesized that modelling catch-up growth (CUG) as developed for coeliac disease (CD), might also fit CUG in adequately treated children with juvenile hypothyroidism (JHT) or growth hormone deficiency (GHD).

**Methods:**

We used a monomolecular function for all available prepubertal data on height standard deviation score (HSDS) minus target height SDS (adjHSDS) in children with JHT (n=20) and GHD (n=18) on a conventional (CoD) or high GH dose (HD), based either on a national height reference with an age cut-off of 10 (girls) and 12 (boys) years (model 1) or prepubertal height reference values, if age (0) was ≥3, with no upper age limit (model 2).

**Results:**

The models could be fitted in 83-90% of cases; in other cases the HSDS decreased after several measurements, which violated the assumption of an irreversible growth process. In JHT, the rate constant (k) and adjHSDS (0) were lower than in CD (p=0.02), but adjHSDS (end) was similar. In GHD (model 1), k was lower than for CD (p=0.004) but similar to JHT, while adjHSDS (0) and adjHSDS (end) were similar to CD and JHT. Thus, the shape of CUG is similar for children with JHT and GHD, while children with CD had less growth deficit at start and a faster CUG. The differences in CUG parameters between GH dose subgroups did not reach statistical significance.

**Conclusion:**

Modelling CUG of prepubertal children with JHT and GHD can be used for assessing the adequacy of CUG and the influence of clinical treatment modalities on its speed and magnitude.

**What is already known on this topic?**

Catch-up growth (CUG) occurs if a growth disorder can be adequately treated. In prepubertal children with coeliac disease treated with a gluten-free diet, height standard deviation score during CUG after start of treatment can be modelled with a monomolecular function.

**What this study adds?**

CUG in most children treated for juvenile hypothyroidism or growth hormone deficiency can be modelled with a monomolecular function. Theoretically, this method may be superior to current outcome parameters to objectify the influence of clinical factors on CUG in growth hormone treated children with growth hormone deficiency.

## Introduction

One of the most fascinating phenomena in the field of regulation of linear growth is catch-up growth (CUG). CUG is a physiological condition of temporary overgrowth, first described by Prader et al (1). In a review paper, the classical form of CUG [type A according to Tanner (2)] was defined as “a height velocity above the statistical limits of normality for age and/or maturity during a defined period of time, following a transient period of growth inhibition” (3). Based on previous studies, CUG usually takes 3-4 years, the duration being dependent on the initial height deficit. The effect of CUG is to take the child toward or right onto his original pre-retardation growth curve (3). On average, this growth curve would be expected to come close to the gender-adjusted midparental height [target height (TH)] standard deviation score (SDS) because, based on twin studies, the genetic influence on adult height (AH) is estimated at approximately 80% (4).

In conditions where the cause of growth failure can be completely compensated or cured [such as hypothyroidism, coeliac disease (CD) and successful operation of an ACTH-secreting pituitary adenoma], one would expect a classical type A CUG. In fact, this has been observed in cohorts and case reports of children with these conditions, including juvenile hypothyroidism (JHT) (5,6). In prepubertal children with JHT (6) CUG is usually complete, but not so in adolescents, probably because of bone age advancement due to simultaneous occurrence of puberty (5). In children with a virtually certain diagnosis of GHD, the growth response to an adequate substitution dosage of GH is expected to have a similar shape and duration as CUG in other forms of secondary growth disorders (7). However, so far in GHD the growth response has usually been expressed as yearly height velocities [cm/year or delta height SDS (HSDS)] (8,9,10), which do not offer an impression of the full pattern of CUG.

We reasoned that, in theory, a mathematical model of the whole phase of CUG would be better than yearly height velocities to assess the adequacy of CUG and to analyse the influence of baseline and treatment-related variables on the growth response to GH treatment in prepubertal GHD children. For prepubertal children with CD, our group (11) reported that HSDS during CUG can be modelled by a monomolecular function: A*(1-B*EXP(-k*t))-5, with t=time in years (0=start of therapy), A-5 = HSDS(end), A*(1-B)-5 = HSDS(0), B = integration constant, and k as rate constant.

For this study we hypothesized that: 1) the monomolecular function developed for CD gives a good fit for CUG in L-thyroxine treated prepubertal children with JHT; 2) the same model can be used for prepubertal children with GHD; and 3) in children with GHD the model can be used to analyse the influence of GH dose on CUG.

## Methods

For this study we used two sets of published data on CUG. The first set was derived from the publication on a retrospective study in German children with JHT (6). For the present analysis we used the individual data as reported in the publication. The second set was derived from a previous publication on a prospective, multicentre, dose-response study in Dutch children with GHD (12). For the present analysis we used the raw data (courtesy Dr. T.C.J.Sas).

All available prepubertal HSDS data were collected, and adjusted for TH (TH, the sex-adjusted mid-parental height). HSDS minus TH SDS, was abbreviated as adjHSDS. From children with JHT (n=20), prepubertal data on yearly adjHSDS for three years were used as reported in the paper (6), and the difference between adjHSDS and adjusted AHSDS (adjAHSDS) was calculated (n=11). HSDS was expressed using the 1966 UK reference data (13,14) and TH was calculated as the sex-adjusted arithmetical mean of parental heights transformed into SDS (13,14). For our analysis, we used an age cut-off of 10 and 12 years for girls and boys, respectively, in order to prevent distorting effects of increasing percentages of pubertal children on mean and SD of height for age in the general population from these ages.

From children with GHD participating in a GH dose-response study (12), all prepubertal data on adjHSDS were used. In this previous study the long-term effect of a conventional dose of GH (0.67 mg/m^2^ body surface per day, n=10, CoD) was compared with a high dose (HD) (1.33 mg/m^2^ body surface per day, n=9, HD). These dosages are approximately equivalent to 24 and 48 ug/kg/day. From the anonymous database containing all data on age, height and pubertal stage the relevant data were selected (courtesy Dr. T.C.J.Sas). As mentioned in this paper (12), the protocol was approved by the medical ethics committees of all participating centres, and all parents gave their written informed consent for the study (coordinating centre: University Medical Centre Rotterdam, registration number: 87.74). For this group, the TH was defined as ½ x (height father + height mother + or - 13) + 4.5 for boys and girls, respectively, because the secular trend over 30 years in the Netherlands was estimated at 4.5 cm between 1965 and 1997 (15).

### Statistical Analysis

For these groups, we took two approaches. First, we used cross-sectional Dutch references (15) with an age cut-off of 10 and 12 years for girls and boys, respectively (model 1), similarly to the approach for JHT. Second, in order to maximize the number of data points and statistical power, we used the IC component of the Infancy-Childhood-Puberty model of longitudinal growth (16) with no age cut-off (model 2).

We modelled all available prepubertal adjHSDS data with a mixed-effects model using a monomolecular function of adjHSDS over time: A*(1-B*EXP(-k*t))-5, with t=time in years (0=start of therapy), A-5 = adjHSDS(end), A*(1-B)-5 = adjHSDS(0), B = integration constant and k as rate constant.

In mathematical terms, this is described as follows:

Let *n* be the number of children, *t* the time in years (0=start of treatment) and *y* the adjHSDS of the *i*-th child at time *t* with *i=1,…,n.* According to the monomolecular growth function the *y* of the *i*-th child can be modelled by the non-linear mixed-effects procedure as:

y_i_(t)=*HSDS*_i_(t)–*THSDS*_i_=*A*(1–Bexp{–*kt*})–5+ε_it_

*with A=A_0_ + A_i0_, B=B_0_+B_i0_, k=k_0_+k_i0_*, with A_0_, B_0_,k_0_ fixed effects and A_i0_, B_i0_, k_i0 _random effects.

The measurement errors ε_it_ are assumed to be independent across individuals and to be normally distributed with mean zero and a common variance. We assume that the random effects have a multivariate normal distribution with mean vector zero and are independent of the measurement errors.

Since by definition CUG has an upward pattern, the model that was chosen for CUG in CD did not allow for a decreasing HSDS (11). In some of our patients a slight downward pattern was noted at the end of CUG. Patients in whom this downward trend was >0.15 SD were excluded from further analysis because a decreasing HSDS (after several measurements showing an increasing HSDS) would violate the assumption of an irreversible growth process.

Modelled CUG was compared between groups (JHT, GHD and CD). To investigate the influence of GH dose on CUG, linear regression analyses were performed to test the difference of the parameters of the monomolecular function between dose groups.

## Results

### Juvenile Hypothyroidism

**Figure 1:**
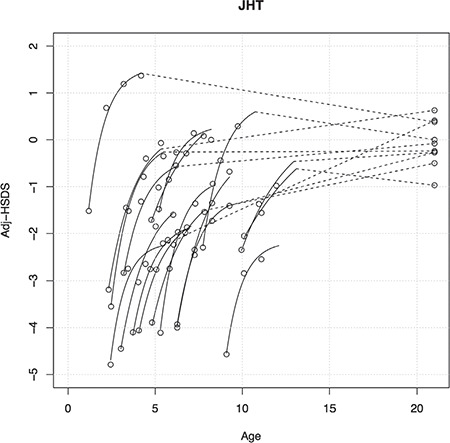
Modelled curves (uninterrupted lines) and raw data (open circles) describing catch-up growth [adjusted height standard deviation score (HSDS) versus age] for each child with juvenile hypothyroidism before reaching puberty, as well as adjusted adult HSDS. Stippled lines connect the last measurement before onset of puberty with adjusted adult HSDS JHT: juvenile hypothyroidism, Adj-HSDS: adjusted-height standard deviation score

In 18 out of 20 cases (90%) of JHT, adjHSDS could be modelled properly. Figure 1 shows the results versus age, and for 11 cases also adjAHSDS. In the 10 out of 11 cases in whom data were available on HSDS after three years of start therapy, mean adjAHSDS was identical to adjHSDS after three years of start therapy, but with a remarkably wide range (-2.6 to 1.9). Thus, in some patients adjAHSDS was substantially lower than adjHSDS after three years, while in others CUG apparently continued after pubertal onset.

**Figure 2:**
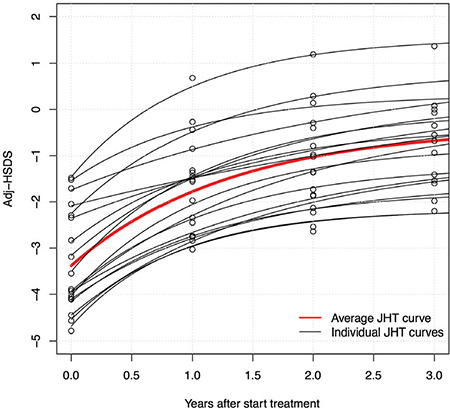
Modelled individual curves and raw data describing catch-up growth of prepubertal children with juvenile hypothyroidism during three years, as well as the average curve JHT: juvenile hypothyroidism, Adj-HSDS: adjusted-height standard deviation score

Figure 2 shows the individual modelled curves of CUG versus time after start of medication, as well as the average CUG curve. Results of the model and the derived adjHSDS at start [adjHSDS(0)] and end of CUG [adjHSDS(end)] are shown in Table 1.

**Suppl Figure 1:**
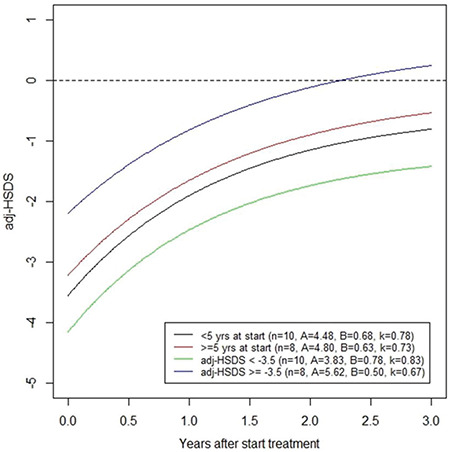
Modelled mean adjusted-height standard deviation score (adj-HSDS) of children with juvenile hypothyroidism in the first 3 years of L-thyroxine therapy, according to age at start (<5 or ≥5 years) and to adj-HSDS at start of therapy (<-3.5 or ≥-3.5). Values for the parameters A, B and k are indicated

The univariate correlations between the rate constant k versus age and adjHSDS(0) were -0.45 (p=0.06) and -0.31 (p=0.21), respectively. Although these correlations are not statistically significant, this implies that the rate constant becomes smaller when age or adjHSDS at start are higher, as illustrated in Suppl Figure 1.

### Growth Hormone Deficiency

**Figure 3:**
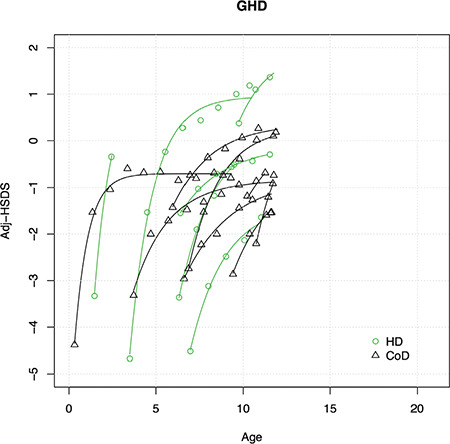
Modelled curves and raw data describing catch-up growth for each child with growth hormone deficiency before reaching puberty Adj-HSDS: adjusted-height standard deviation score, GHD: growth hormone deficiency, HD: high dose, CoD: Conventional dose

**Figure 4:**
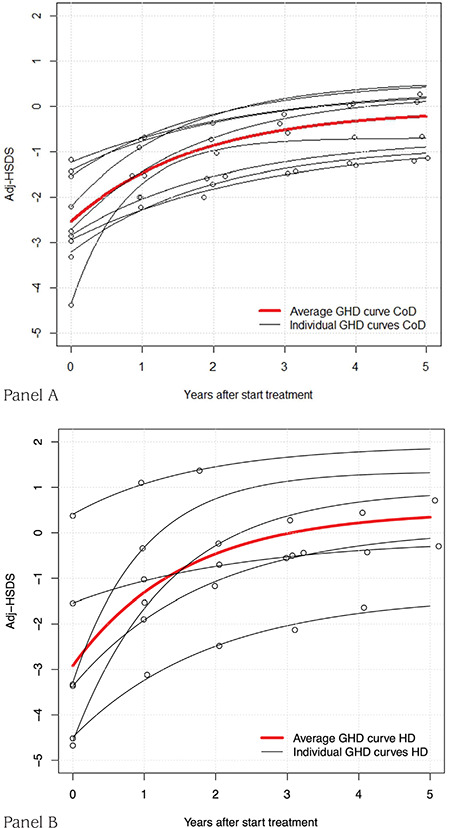
Modelled individual curves and raw data describing catch-up growth of prepubertal children with growth hormone deficiency during three years, as well as the average curve. Panel A: conventional growth hormone dose. Panel B: high growth hormone dose Adj-HSDS: adjusted-height standard deviation score, GHD: growth hormone deficiency, HD: high dose, CoD: Conventional dose

In 15 out of 18 cases (83%) with GHD adjHSDS could be modelled properly (Figure 3). Figure 4 shows the individual modelled curves of CUG versus time after start of medication, as well as the average CUG curve, in both dose groups (panels A and B). Results of the derived adjHSDS(0) and adjHSDS(end) of CUG for both models are shown in Table 1. There was a tendency toward a faster CUG (k) and higher adjHSDS(end) in the HD group compared to CoD, but the difference did not reach statistical significance (p=0.626 and 0.293 in models 1 and 2, respectively). After correction for adjHSDS at start, the difference was 0.04 (p=0.772, model 1) and 0.07 (p=0.228, model 2). The difference between adjHSDS(end) in the CoD and HD groups was 0.56 (p=0.326) and after adjustment for age and adjHSDS at start 0.68 (p=0.108) using model 1. For model 2, these were 0.54 (p=0.428) and after adjustment for age and adjHSDS at start 0.67 (p=0.189), suggesting that model 2 may be more sensitive to detect effects of clinical parameters than model 1.

### Comparison Between Diagnostic Groups

**Figure 5:**
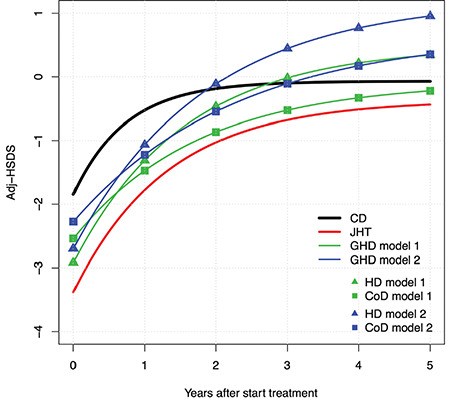
Modelled mean adjusted-height standard deviation score of children with juvenile hypothyroidism, growth hormone deficiency (CoD and high dose, models 1 and 2) in comparison with the catch-up growth model for celiac disease Adj-HSDS: adjusted-height standard deviation score, CD: celiac disease, HD: high dose, GHD: growth hormone deficiency, JHT: juvenile hypothyroidism, CoD: Conventional dose

The modelled mean adjHSDS of JHT, GHD (models 1 and 2) and CD is shown in Figure 5. Compared with CD, in JHT adjHSDS at start was lower (p=0.0002) as well as k (p=0.02), also after adjustment for adjHSDS(0) (p=0.003), but adjHSDS after three years was equal (Table 1). In GHD patients, using model 1, k was lower than for CD but similar to JHT; adjHSDS(0) and adjHSDS(end) were similar to CD and JHT.

Table 2 shows the predicted growth parameters of the monomolecular function given adjHSDS at start. This information could be useful in predicting the growth trajectory at start of treatment and monitoring specific treatment cases. For example, if a child with JHT has an adjHSDS of -3 at start of treatment, the predicted growth trajectory could be described by: adjHSDS (t)=4.99*(1-0.60*EXP(-0.72*t))-5, with t in years. The predicted adjHSDS two years after start treatment (t=2) is then expected to be -0.72.

## Discussion

The shape of CUG in most children treated for JHT and GHD, when expressed as HSDS or adjHSDS, can be described using the same monomolecular model as we reported for CD (11). The average curves of CUG in JHT and GHD were similar, and differed from the model for CD in the sense that the rate constant was lower (thus a less fast CUG) and the end of CUG was reached later, which is probably related to more initial height deficit than in patients with CD. Advantages of modelling CUG in comparison to yearly indicators of growth include that a full picture is obtained of CUG by using all available growth data rather than data at full years. An additional advantage of this procedure is that measuring errors are smoothed out. A potential adaptation of our approach is to analyse the effect of various predictors (before and during treatment) on the whole phase of CUG in children with GHD. One could, for example, envisage that this technique might be more sensitive to detect additional predictors of the growth response than the ones discovered in the studies by Ranke et al (8) and Ranke and Lindberg (17) using first and second year height velocity. Regarding the effect of variables affecting the growth response to GH during treatment, we recently demonstrated the usefulness of this approach by reporting on the effect of various degrees of non-adherence on CUG in GHD patients included in a large database (18).

CUG occurs after the initiation of appropriate treatment of growth impairment due to various conditions, including endocrine disorders (hypothyroidism, Cushing syndrome, GHD), gastrointestinal diseases (CD), and psychosocial disturbances (psychosocial deprivation and starvation with psychosocial deprivation). In prepubertal children, the phase of CUG is followed by a maintenance phase, in which HSDS remains stable, as shown for GHD (7). In adolescence, linear growth (thus also CUG) is strongly influenced by the timing of puberty, so that during this phase CUG cannot be analysed separately. Therefore, during this phase the effect of a certain treatment on growth is usually expressed as total pubertal height gain (19).

Tanner (2) distinguished three types of CUG: type A, B and C. Type A is the classical pattern of CUG, which takes the child back onto his original pre-insult centile or SDS position within a few years. Types B and C are characterized by a normalization of height velocity for bone age (type B) or age (type C), in combination with a delayed maturation, which in the end may result in a normal AH. More recently, we proposed an intermediate type AB, in which CUG initially does not result in complete normalization of HSDS, but still leads to a normal AH because of delayed maturation (20). Such a pattern was particularly evident in patients with GHD on a relatively low GH dose, as illustrated by several individual curves of patients participating in the dose-response study (12), as shown in Figures 3 and 4. It therefore appears that there is a difference between CUG in GHD versus conditions where the cause can be removed completely (e.g. hypothyroidism, removal of tumour, etc): in GHD CUG is dependent on GH dose, and in each individual child it is impossible to know what the GH dose should be to mimic the “natural” GH secretion during the various phases of CUG observed in children with other conditions.

The mechanism responsible for CUG is still elusive. For the neuroendocrine hypothesis proposed by Tanner in 1963 (21) the experimental evidence has not been convincing so far. In line with the growth plate hypothesis of Baron et al (22), based on earlier work of Williams et al (23,24), Emons et al (25) observed that CUG in infants with CD showed a pattern of normal growth velocity for height age, a pattern described by Tanner as type B CUG (2). However, we believe that this hypothesis cannot fully explain the very fast initial height velocity (much faster than normal for bone age) that can be observed in older children with CD (26) as well as in some children with JHT and GHD (illustrated by some individual curves in the present study). More recently, additional pathophysiologic mechanisms have been proposed, for example regarding a possible role of ghrelin (27), sirtuins, fibroblast growth factor 21, and specific miRNAs and histone deacetylases, reviewed in (28).

According to the current definition of CUG (3), we previously proposed that the growth response to GH in non-GHD patients should not be called “CUG”, but should rather be termed “therapy-induced growth enhancement” (29). It would be interesting to analyse to what extent the mathematical model of CUG that we developed for CD, JHT and GHD applies to the growth pattern of GH-treated children with non-GHD conditions, such as children born small-for-gestational age with failure to catch-up spontaneously after birth, Turner syndrome and idiopathic short stature.

### Study Limitations

We acknowledge that the number of patients in both patient groups is limited, and that the restriction that CUG can only be properly studied in prepubertal children further limits the number of data that could be used for the analysis. We tried to alleviate this restriction by using a prepubertal growth reference (model 2), whereby the number of measurements could be maximized. This indeed led to lower p-values in the comparison between GH dosage groups, but we assume that the considerable variation in CUG curves between individuals and the relatively small patient groups precluded reaching statistical significance. Therefore, this report should be considered rather as a proof of principle than as a definitive study. Further, the variation in the pattern of CUG in both diagnostic groups is striking. In particular, the apparent “overshoot” of CUG in some patients with JHT in contrast to insufficient CUG before puberty in others is difficult to explain. Similarly contrasting CUG patterns were seen in children with GHD, and in these children our data suggest that an overshoot of prepubertal CUG was seen more often in children treated with the high GH dose than on a conventional dose, as reported previously (12). However, the low number of patients with sufficient prepubertal data and the uncertainty about AH in the GH dose-response study precludes a firm conclusion.

## Conclusion

CUG of prepubertal children with CD, JHT or GHD can be modelled with a monomolecular function. This can be used for assessing the adequacy of CUG and the influence of pretreatment variables, GH dose and adherence on the growth response to GH in prepubertal GHD children.

## Figures and Tables

**Table 1:**
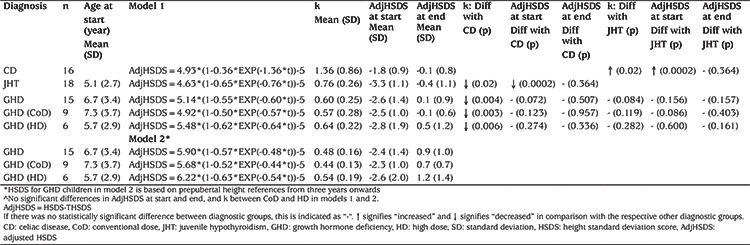
Monomolecular models for celiac disease, juvenile hypothyroidism and growth hormone (GH) deficiency treated with conventional (CoD) or high GH dose^

**Table 2:**
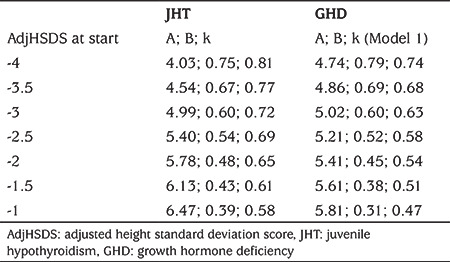
Predicted growth parameters of the monomolecular function given adjusted height standard deviation score at start
